# Chimeric VLPs Bearing VP60 from Two Serotypes of *Rabbit Haemorrhagic Disease Virus* Are Protective against Both Viruses

**DOI:** 10.3390/vaccines9091005

**Published:** 2021-09-09

**Authors:** Kevin P. Dalton, Carmen Alvarado, Edel Reytor, Maria del Carmen Nuñez, Ana Podadera, Diego Martínez-Alonso, Jose Manuel Martin Alonso, Ines Nicieza, Silvia Gómez-Sebastián, Romy M. Dalton, Francisco Parra, José M. Escribano

**Affiliations:** 1 Instituto Universitario de Biotecnología de Asturias, Departamento de Bioquímica y Biología Molecular, Campus El Cristo, Universidad de Oviedo, Edificio Santiago Gascón, 33006 Oviedo, Spain; daltonkevin@uniovi.es (K.P.D.); anapoda@uwindsor.ca (A.P.); jmmartin@uniovi.es (J.M.M.A.); inesnila@gmail.com (I.N.); fparra@uniovi.es (F.P.); 2 Alternative Gene Expression S.L. Ronda de Poniente 14, Tres Cantos, 28760 Madrid, Spain; calvarado@algenex.com (C.A.); edel.reytor@avantorsciences.com (E.R.); cnunez@algenex.com (M.d.C.N.); diegomartinezalonso91@gmail.com (D.M.-A.); sgosebastian@gmail.com (S.G.-S.); romydalton@algenex.com (R.M.D.)

**Keywords:** calicivirus, RHDV, chimeric VLP, bivalent vaccine, *Trichoplusia ni*, baculovirus

## Abstract

The VP60 capsid protein from rabbit haemorrhagic disease virus (RHDV), the causative agent of one of the most economically important disease in rabbits worldwide, forms virus-like particles (VLPs) when expressed using heterologous protein expression systems such as recombinant baculovirus, yeasts, plants or mammalian cell cultures. To prevent RHDV dissemination, it would be beneficial to develop a bivalent vaccine including both RHDV GI.1- and RHDV GI.2-derived VLPs to achieve robust immunisation against both serotypes. In the present work, we developed a strategy of production of a dual-serving RHDV vaccine co-expressing the VP60 proteins from the two RHDV predominant serotypes using CrisBio technology, which uses *Tricholusia ni* insect pupae as natural bioreactors, which are programmed by recombinant baculovirus vectors. Co-infecting the insect pupae with two baculovirus vectors expressing the RHDV GI.1- and RHDV GI.2-derived VP60 proteins, we obtained chimeric VLPs incorporating both proteins as determined by using serotype-specific monoclonal antibodies. The resulting VLPs showed the typical size and shape of this calicivirus as determined by electron microscopy. Rabbits immunised with the chimeric VLPs were fully protected against a lethal challenge infection with the two RHDV serotypes. This study demonstrates that it is possible to generate a dual cost-effective vaccine against this virus using a single production and purification process, greatly simplifying vaccine manufacturing.

## 1. Introduction

Rabbit haemorrhagic disease virus (RHDV) is the causative agent of a highly infectious and fatal disease affecting domestic and wild rabbits [1,2,3]. It is considered to be the single most economically important disease in rabbits worldwide with serious economic effects on cuniculture [4]. RHDV is a non-enveloped, icosahedral RNA virus, belonging to the *Lagovirus* genus within the *Caliciviridae* [5]. The positive-sense single-stranded RNA genome of RHDV is 7.5 kb in length and encodes a polyprotein precursor from the open reading frame (ORF) 1, which is finally cleaved into the 60 kDa major capsid protein (VP60, also known as VP1) and several non-structural proteins [6,7]. Additionally, VP60 may also be expressed from a subgenomic RNA of approximately 2.4 kb [8,9]. The virus capsid (approx. 40 nm in diameter) comprises 180 copies (90 dimers) of VP60, arranged with T = 3 symmetry to form 12 pentamers and 20 hexamers [10].

Commercially available vaccines are still traditional inactivated forms of the original pathogens [11], which are produced from livers collected from artificially infected rabbits. Although highly effective for controlling RHD, these types of vaccines often raise safety concerns because of the potential for the spread of the virus during the process of viral inactivation [12] and their production is ethically questionable. Virus-like particles (VLPs) are supramolecular assemblages with well-defined geometry that mimic the overall structure of native virions, while lacking any viral genome. They are composed of multiple copies of one or more viral proteins and are usually antigenically indistinguishable from an infectious virus or subviral particles, having been recognised as safe and effective vaccine candidates that simultaneously lead to strong humoral and cellular immune responses [13,14,15,16].

In the course of its evolution, RHDV has thus far diverged to form six recognised genotypes [17], all highly pathogenic and virulent. Genotype 6 is the antigenic subtype (RHDVa) that became prevalent in certain countries, including the USA [18]. Using the proposed nomenclature for lagoviruses in this genus, these genogroups are now termed RHDV GI.1a-d/ [19] with RHDVAST89 a reference sequence for RHDV GI.1. In 2011, outbreaks of a new variant (originally termed RHDVb/RHDV2) causing mortalities were reported on Spanish rabbit farms [20,21]. This variant appears to be closely related to a French isolate described in 2010 [22]. Inactivated vaccines derived from all RHDV variants are not cross-protective. In light of this and the immunogenic differences at the serotype level, it is important to develop bivalent vaccines to obtain wide-range protection of farmed rabbits [23].

The VP60 capsid protein expressed in heterologous recombinant systems forms characteristic VLPs, which are protective in immunised rabbits. VP60-derived VLPs have been obtained in *Escherichia coli* [24], *Saccharomyces cerevisiae* [25], plants [26,27], and mammalian and insect cells using recombinant poxvirus [28,29,30] and baculovirus vectors, respectively [12,31,32,33,34]. However, to date, none of the vaccines targeting RHDV have been successfully commercialised. We therefore aimed to develop a dual RHDVGI.1/RHDVGI.2 vaccine using the Baculovirus Expression Vector System (BEVS) using CrisBio technology, based on the use of *Trichoplusia ni* pupae as natural bioreactors [35] to increase vaccine production efficiency in a cost-effective manner. In order to reduce the downstream production-associated costs, our strategy was to generate chimeric VLPs containing the VP60 proteins from the two serotypes obtained by co-infection of the insect pupae with two recombinant baculoviruses expressing either capsid protein. The resulting chimeric VLP assembled T = 3 stable structures containing copies of the two VP60 proteins, and when used to immunise rabbits, it conferred protection against a lethal dose challenge with a RHDV GI.1 and RHDV GI.2 virulent viruses.

## 2. Materials and Methods

### 2.1. Insect Cell Culture and Baculoviruses

The *Spodoptera frugiperda* Sf21 cell line was cultured at 28 °C in TNM-FH culture media (PAN-Biotech GmbH, Aidenbach, Germany) supplemented with 10% heat-inactivated foetal bovine serum (PAN-Biotech GmbH, Germany), gentamicin at 50 µg/mL (Sigma, St. Louis, MO, USA (Merck)), and Antibiotic-Antimicotic 1× (Sigma, St. Louis, MO, USA (Merck)). Recombinant baculoviruses (rBacs) were generated using the TopBac expression cassette [36,37] as donor plasmids to generate the bacmids with the Bac-To-Bac baculovirus expression system (Invitrogen, Thermo Fisher Scientific, Waltham, MA, USA). Bacmids were transfected into Sf21 cells using Cellfectin^®^ II Reagent (Thermo Fisher Scientific) and following the manufacturer’s instructions.

Baculovirus stocks were titered on Sf21 cells using the 6-well plate format. Subconfluent Sf21 cells were infected with 10-fold serial dilutions of recBac stocks for 1 h at 28 °C. Subsequently, inocula were removed and cells were overlayed with an insect cell media-agarose mix at 1% agarose final concentration (UltraPure™ Low Melting Point Agarose, Life Technologies, Carlsbad, CA, USA) and further incubated for 3–5 days. Monolayers were stained with neutral red to facilitate plaque counting.

### 2.2. Construction of Recombinant Baculoviruses

The improved baculovirus vector system TopBac was used for the generation of recombinant baculoviruses. The construction of recombinant baculoviruses was carried out essentially as described in [36], with the addition of the generation of the transfer vector containing the RHDV VP60-encoding genes. Gene sequence synthesis was carried out using the GenScript (Leiden, The Netherlands) service. The protein sequences encoded by the genes were the following:

RHDV GI.1 Isolate AST89 Genbank accession number Z29471

MEGKARTAPQGEAAGTATTASVPGTTTDGMDPGVVATTSVVTAENSSASIATAGIGGPPQQVDQQETWRTNFYYNDVFTWSVADAPGSILYTVQHSPQNNPFTAVLSQMYAGWAGGMQFRFIVAGSGVFGGRLVAAVIPPGIEIGPGLEVRQFPHVVIDARSLEPVTITMPDLRPNMYHPTGDPGLVPTLVLSVYNNLINPFGGSTSAIQVTVETRPSEDFEFVMIRAPSSKTVDSISPAGLLTTPVLTGVGNDNRWNGQIVGLQPVPGGFSTCNRHWNLNGSTYGWSSPRFADIDHRRGSASYPGSNATNVLQFWYANAGSAIDNPISQVAPDGFPDMSFVPFNGPGIPAAGWVGFGAIWNSNSGAPNVTTVQAYELGFATGAPGNLQPTTNTSGSQTVAKSIYAVVTGTAQNPAGLFVMASGVISTPSANAITYTPQPDRIVTTPGTPAAAPVGKNTPIMFASVVRRTGDVNATAGSANGTQYGTGSQPLPVTIGLSLNNYSSALMPGQFFVWQLTFASGFMEIGLSVDGYFYAGTGASTTLIDLTELIDVRPVGPRPSKSTLVFNLGGTANGFSYV*

RHDV GI.2—previously RHDVb/2 isolate Nav10/11 accession number KM878681

MEGKARTASQGETAGTATTASVPGTTTDGMDPGVVATTSVVTTENASTSIATAGIGGPPQQVDQQETWRTNFYYNDVFTWSVADAPGNILYTVQHSPQNNPFTAVLSQMYAGWAGGMQFRFIVAGSGVFGGRLVAAVIPPGIEIGPGLEVRQFPHVVIDARSLEPVTITMPDLRPNMYHPTGNPGLVPTLVLSVYNNLINPFGGSTSAIQVTVETRPSEDFEFVMIRAPSSKTVDSISPADLLTTPVLTGVGTDNRWNGEIVGLQPVPGGFSTCNRHWNLNGSTFGWSSPRFAAIDHDRGNASYPGSSSSNVLELWYASAGSAADNPISQIAPDGFPDMSFVPFSGTTVPTAGWVGFGGIWNSSNGAPFVTTVQAYELGFATGAPSNPQPTTTTSGAQIVAKSIYGVATGINQATAGLFVMASGVISTPNSSAITYTPQPNRIVNAPGTPAAAPIGKNTPIMFASVVRRTGDINAEAGSTNGTQYGAGSQPLPVTVGLSLNNYSSALMPGQFFVWQLNFASGFMELGLSVDGYFYAGTGASATLIDLSELVDIRPVGPRPSTSTLVYNLGGTTNGFSYV*

The recombinant baculoviruses produced were subjected to two rounds of clonal selection by plaque formation, and after virus propagation, infective titres of approximately 10^8^ pfu (plaque-forming units)/mL were reached. Baculovirus stocks were stored at −80 °C containing 5% glycerol. The correct expression of VP60 proteins from recombinant baculoviruses was confirmed by detection of proteins using specific sera by Western blot analysis.

### 2.3. Production of Recombinant Proteins in Insect Pupae

*Trichoplusia ni* (*T. ni*; Cabbage looper) insects were reared following a previously described methodology [35]. Briefly, eggs were placed into larvae developmental cages, containing an artificial insect diet. The eggs hatched and larvae proceeded through the 5 instar stages and then pupated. Silk was then removed from the pupae, and each was automatically injected using an inoculation robot with 5 µL of recombinant baculovirus diluted to reach the number of pfu per dose selected, collecting the pupae 5 days after infection. The pupae collected at each time point were weighed to obtain the total biomass and were frozen immediately to be stored at −20 °C until they were processed for recombinant protein extraction. The total productivity yields for each condition was analysed and compared by SDS-PAGE and densitometry of the corresponding band.

### 2.4. Protein Recovery from Infected Pupae

The crude protein extracts were obtained by homogenising the frozen biomass in the adequate protein extraction buffer. The resulting suspension was subjected to the downstream processing [35] to obtain the final antigen product, where the amount of recombinant protein was quantified by densitometry on SDS-PAGE using a BSA standard curve. Co-infection with both baculoviruses was carried out using different pfu of each virus to optimise the chimeric VLP formation. The samples were stored at 4 °C until they were used to formulate the vaccine and immunise the animals with the different doses.

### 2.5. Electron Microscopy Analysis of VLP

Electron microscopy analyses were performed by conventional means. Briefly, purified VLPs (approximately 5 µL) were applied to glow-discharged carbon-coated grids for 2 min. Samples were negatively stained with 2% (*w*/*v*) aqueous uranyl acetate. Micrographs were recorded with an EM 2000 Ex microscope (JEOL, Tokyo, Japan). For the quantitation analysis of VLP stability, samples obtained from GI.2 (N11-) and GI.1 (AST) infections or from co-infections with the two viruses under different conditions were analysed at day 0 and 60 of storage at 4 °C. Samples were diluted to a protein concentration of 25 mg/mL to obtain an even distribution of the capsids on the electron microscopy grid and an easily countable number of capsids per image at the suitable magnification. Three grids were prepared per sample and images were taken in four different areas of each grid. We quantified the capsids with ImageJ tools in 15 pictures.

### 2.6. Dot Blot Analysis of Chimeric VLPs

Monoclonal antibodies were absorbed to a nitrocellulose membrane and thenblocking VLPs were bound to the Mab-coated membrane. Following this, VLP binding was determined by specific peroxidase labelled 2D9 Mab binding. In this format, 1 µL per dot containing the Mab dilution was added on the nitrocellulose membrane. The drop was allowed to air dry for 15 min before the blocking solution (5% milk powder dissolved in PBS) was added. All washing steps were carried out in triplicate using PBS-Tween (0.05% PBS-Tw). For peroxidase-labelled antibodies, the Pierce™ ECL Western Blotting Substrate system was used for detection (Thermo Fisher Scientific).

### 2.7. ELISAs for the Detection of Chimeric VLPs

ELISA were carried out in flat bottomed 96-well plates (Nunc™ MaxiSorp, Thermo Fisher Scientific). Plates were coated with 100, 50, or 10 ng of Mab (varying concentrations are indicated in the results section) in each well in PBS (1 mM a pH 7.3 with sodium azide 0.05% PBS-AS). Coating plates were incubated overnight at 4 °C. The next day the supernatant was removed, and plates were blocked with 1% BSA in PBS-AS for 1 h at 37 °C. Plates were washed 3 times with 200 µL PBS-Tw per well. Antibodies were added in volumes of 100 µL per well and incubated for 1 h at 37 °C followed by 3 washing steps as previously described. Secondary antibodies at the indicated dilutions were incubated for 1 h at 37 °C. Prior to revelation, plates were washed 3 times in PBS-Tw. For reaction development TMB (3, 3′, 5, 5′-tetrametilbenzidina) (Sigma) (100 µL per well) was added and incubated at room temperature in the dark until the reaction was stopped using 100 µL 2M H_2_SO_4_. Absorbances were read at 450 nm using Varioskan Flash microplate reader and Skanit software.

### 2.8. Experimental Challenge and Ethical Considerations

New Zealand White rabbits were supplied by San Bernardo Farm (Navarra, Spain). Prior to vaccination, sera were collected from the marginal ear vein of all animals to confirm the absence of RHDV antibodies.

Two protection experiments were carried out. In general terms, rabbits were vaccinated with chimeric RHDV VLPs and in experiment 1; 7 days post vaccination they were challenged with RHDV GI.2 and in experiment 2; 7 days post vaccination they were challenged with classic RHDV GI.1 isolate RHDV AST89.

Experimental challenge 1: 30-day-old rabbits were divided into 4 groups (Groups A–D), each of 5 animals. Each group was vaccinated with the following VLPs administered in a total volume of 0.5 mL:

Group A: Non-vaccinated controls.
Group B: 20 µg chimeric VLPs.
Group C: 40 µg chimeric VLPs.
Group D: 5 µg RHDV G1 VLPs + 5 µg RHDVb VLPs.

Experimental challenge 2: 2-month-old rabbits were divided into 2 groups (Groups E and F), each of 5 animals. Each group was vaccinated with the following VLPs administered in a total volume of 0.5 mL:

Group E: Non-vaccinated controls.
Group F: 40 µg chimeric VLPs.

Rabbits were kept under observation throughout the experiment and housed in pairs in a biosecurity level 2 laboratory. Table 1 shows a summary of the experimental design.

### 2.9. Virus Isolate Used for Challenge

Two virus isolates were used in this study. Groups A–D were challenged 7 days post vaccination with isolate *L. europaeus* GI.2 virus, common name RHDV-Gal08/13, with 10 LD_50_. Groups E and F were challenged 7 days post vaccination with RHDV isolate *L. europaeus* RHDV GI.1 RHDV-Ast89 (Genbank accession number Z49271). Rabbits were infected on day 7 post vaccination with 150 hemagglutination units/0.5 mL (as determined using O- and B-type human erythrocytes). The virus was administered subcutaneously in the cervical area as a dilution of clarified infected liver homogenates. Euthanasia of surviving rabbits was carried out by intravenous injection with Dolethal following the manufacturer’s instructions (pentobarbital sodium BP 20%, Vetoquinol, S.A, Madrid, Spain).

Symptoms of RHDV infection, such as nervous, respiratory, and digestive signs, were monitored and recorded daily. Blood samples were collected on days 0 and 7 post vaccination, and from all surviving rabbits 7 days post challenge. Serum was separated after clotting by centrifugation and stored at −20 °C until analysis. After euthanasia, rabbits were necropsied, and gross lesions were recorded. Samples of liver were directly frozen at −80 °C for total RNA extraction using the GenElute Mammalian Total RNA Miniprep Kit (Sigma, St. Louis, MO, USA), and RHDV GI.2 VP60 detection and quantification were obtained by specific RT-qPCR [38].

### 2.10. Detection of RHDV Specific Antibodies by ELISA

Rabbit serum RHDV antibody levels were tested by enzyme-linked immunosorbent assay (ELISA) using the commercially available INgezim RHDV kit (Ref: 17.RHD.K1, Ingenasa, Madrid, Spain) following the manufacturer’s instructions. The absorbance was measured at 450 nm in a microplate reader (Varioskan Flash, Thermo Fisher Scientific) and data were analysed using the Skanit software Version 2.4.5 (Thermo Fisher Scientific). Negative and positive control sera were included in all plates. Results are expressed as mean optical densities ± SD (*n* = 5) from each group.

## 3. Results

### 3.1. Expression of VP60 Proteins and VLP Conformation

Different doses of the previously titrated recombinant baculoviruses expressing the VP60 proteins were used to infect pupae batches. The infection procedure consisted in the inoculation of the pupae allocated in disposable pupae plastic trays. These trays, compatible with a specially designed baculovirus inoculation robot, were allocated in the machine and injected with the previously established dose of the recombinant baculovirus vector by a needle connected to a precision pump, which dispenses microliter volumes. After inoculation, the infected pupae were incubated for several days at a previously determined temperature. The optimal VP60 protein production conditions were determined analysing the kinetics of protein synthesis under different conditions based on virus dose and infected pupae incubation times. After the protein production peak was reached, pupae were collected and stored frozen in plastic bags under vacuum until downstream processing.

**Figure 1:**
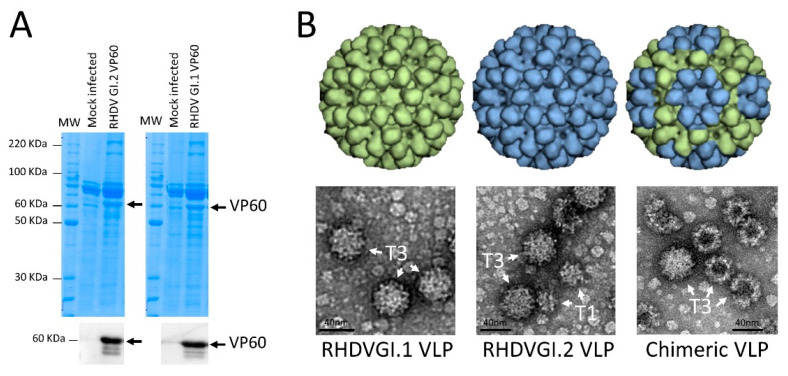
Expression of protein VP60 from RHDV GI.1 (isolate AST89) and RHDV GI.2 (isolate N11) in *T. ni* pupae by two different recombinant baculoviruses, and VLP formation by infecting the pupae with individual baculovirus or co-infecting with both vectors. (**A**) SDS-PAGE gels resolving the extracts from infected pupae with individual baculovirus and stained by Coomassie blue. VP60 protein is indicated with an arrow. VP60 proteins were also detected by Western blot using the monoclonal antibody 1A2 recognising the VP60 from both serotypes. The full uncropped blot is included as supplemental Figure S1. (**B**) A 3-D model of the hypothetic conformation of the resulting VLP after infection with individual recombinant baculovirus and the resulting VLPs after co-infecting with both vectors. Representative electron micrographs of purified VLPs resulting from the infection with individual baculovirus or after a co-infection (chimeric). T3 and T1 structures observed are indicated in the images with arrows.

The VP60 proteins were expressed as a major single band of 60 KDa in extracts resolved in SDS-PAGE gels (Figure 1A). VP60 derived from the GI.1 (AST89) virus showed a slightly higher electrophoretic mobility than VP60 derived from the GI.2 (N11) virus. At the optimal expression conditions, the productivity was on average around 6 mg ± 5% for the GI.1 AST89-derived VP60 and around 7 mg ± 10% for the GI.2 N11-derived VP60 recombinant protein per gram of infected biomass, measured in three independent production batches and calculated by the densitometry of stained gels using a BSA curve. When the proteins were extracted from pupae with a buffer PBS pH 7.2, >90% of the VP60 proteins remained in the soluble fraction of the extracts. To purify the recombinant VP60 proteins, we established a simple protocol of precipitation using ammonium persulfate in order to reduce the insect-derived contaminants. The rest of the downstream process consisted of a tangential flow filtration and an additional filtration step to obtain a sterile vaccine preparation, recovering around 70% with an antigen purity > 80%.

RHDV mature virions are spherical, non-enveloped, icosahedral particles of 32–40 nm in diameter, whose capsid consists of 90 dimers of capsid protein VP60. In order to demonstrate that the pupae-derived VP60 proteins were capable of forming VLPs, we carried out an analysis of the purified VP60 by transmission electron microscopy (negative staining). Figure 1B shows representative images of purified VLPs obtained after infection with the two recombinant baculoviruses. A large number of structures could be seen corresponding in shape and size with the VLP described for the rabbit virus, confirming the viability of the CrisBio technology for the production of RHDV VLPs. Interestingly, while the GI.1 AST89-derived VLPs showed mainly T3 structures, the GI.2 N11-derived VLPs also showed a large proportion of T1 particles. Co-infection experiments with the two VP60-encoding vectors also showed good VLP productivities, and electron micrographs showed more similar ratios between T3 and T1 structures than GI.1-derived VLPs when the ratio of GI.2/GI.1 baculoviruses was 3:1. However, the percentage of T1 structures was slightly higher at a ratio of 1:3 and much higher when used at a 1:1 ratio (data not shown).

**Figure 2:**
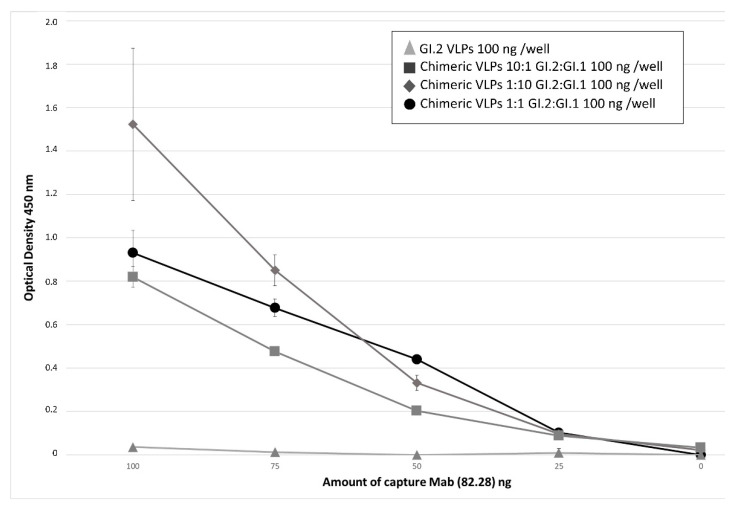
Sandwich ELISA using serotype-specific Mabs for capture and detection of chimeric VLPs. ELISA plate wells were coated with decreasing quantities of GI.1 (AST89)-specific Mab 82.28 (100, 75, 50, or 10 ng). The graph shows mean optical densities (450 nm) ± SD (*n* = 3), Following incubation with GI.2 VLPs or chimeric VLPs produced by co-infection of baculovirus in insect cells at varying ratios (GI.2:GI.1 of 10:1 (grey squares), 1:10 (grey diamonds), and 1:1 (black circles), binding was detected using peroxidase-conjugated GI.2-specific Mab 2D9. Optical density values measured at 450 nm are shown. Positive detection of chimeric VLPs indicates the incorporation of GI.1 and GI.2 VP60 in the same VLPs.

To determine if VLPs obtained after co-infecting with the two baculoviruses were formed by a single VP60 protein or were chimeras of the two VP60 proteins, we designed ELISA and dot blot-based sandwich analyses using monoclonal antibodies capable of recognising one or the two VP60 proteins. Figure 2 shows the results of the ELISA used to detect chimeric VLP formation from insect cells using baculovirus co-infections at three MOI ratios. The ratios were as follows, GI.2:GI.1 1:1, 10:1, and 1:10. As a control, non-chimeric GI.2 VLPs were analysed. The GI.1-specific monoclonal antibody (Mab) 82.28 (A. Podadera, PhD thesis University of Oviedo) was used as a capture antibody in decreasing amounts (100, 50, and 10 ng/well). Subsequently, VLPs were added and 82.28 Mab-VLP binding was detected using a peroxidase-conjugated GI.2-specific Mab 2D9. Control GI.2 VLPs were not recognised by the capture (82.28) Mab (Figure 2) and therefore were not detected by the GI.2-specific 2D9 antibody. VLPs generated by coinfection of GI.2 and GI.1 VP60-expressing baculoviruses both bound the GI.1-specific Mab and were detected by the GI.2-specific Mab 2D9, indicating their chimeric nature. At MOI ratios of 1:1 and 1:10 (GI.2:GI.1), equivalent levels of chimeric VLP binding were detected. At a reduced GI.1 MIO (ratio 10:1 GI.2:GI.1), lower levels of chimeric VLPs were detected, highlighting the specificity of the 82.28 Mab and reduced GI.1 VP60 incorporation (Figure 2) following baculovirus co-infection at this ratio.

**Figure 3:**
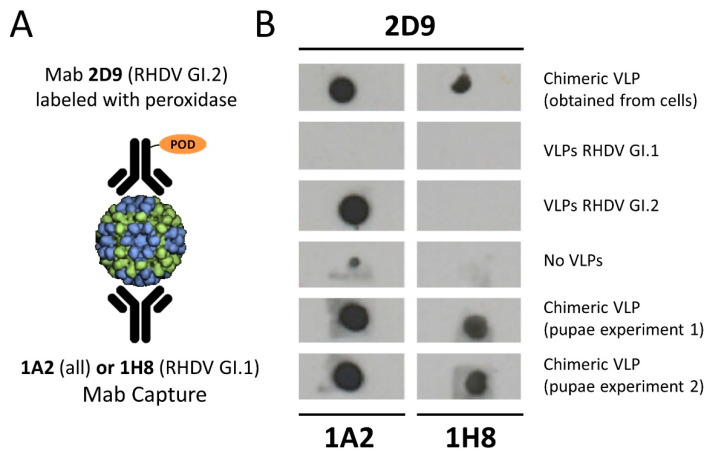
Determination of the incorporation RHDV GI.1- and RHDV Gi.2-derived VP60 proteins into the chimeric VLP by dot blot using different Mabs. (**A**) Design of a capture dot blot using a Mab (1A2) with broad recognition of RHDV serotypes or a RHDV GI.1-specific Mab (1H8) to capture the purified chimeric VLPs. Immune complexes were revealed with an RHDV GI.2-specific Mab (2D9). (**B**) Dot blot results obtained in the different assays. All dot blots were positive for the chimeric VLPs when both capture antibodies were used, indicating the dual VP60 composition of the VLP. Negative results were obtained with the VLP obtained from infections with a single recombinant baculovirus expressing the VP60 from RHDV GI.1 and RHDV GI.2 (captured with Mab 1H8) and control samples. The full uncropped dotblot is included as supplemental Figure S2.

Figure 3A shows the dot blot study design and Figure 3B shows the results obtained. When a Mab recognising both RHDV types was used to capture the purified VLP (Mab 1A2), and the immune complex was developed by a secondary Mab against GI.2 virus, the dot blot was negative both with the GI.1 (AST89)-purified VLP and in the negative control. However, the reaction was positive for the GI.2 (N11) and chimeric-purified VLP. When the VLP capture was carried out with a Mab specific to GI.1 (AST89) (Mab 1H8) and revealed with the GI.2 (N11) Mab, chimeric VLPs showed reactivity. Chimeric VLPs produced in insect cells and recovered from the cell media showed identical results to those observed with the CrisBio-derived VLP (Figure 3B). These results confirmed the presence of both VP60 proteins derived from the GI.1 (AST89) and GI.2 (N11) viruses in the recombinant VLPs obtained by co-infection of the recombinant baculovirus vectors. These results were confirmed in VLPs obtained from two independent batches of infected pupae.

### 3.2. Relevance of Recombinant Baculovirus Ratios Used in Co-Infections on the Stability of the Chimeric VLP

**Figure 4:**
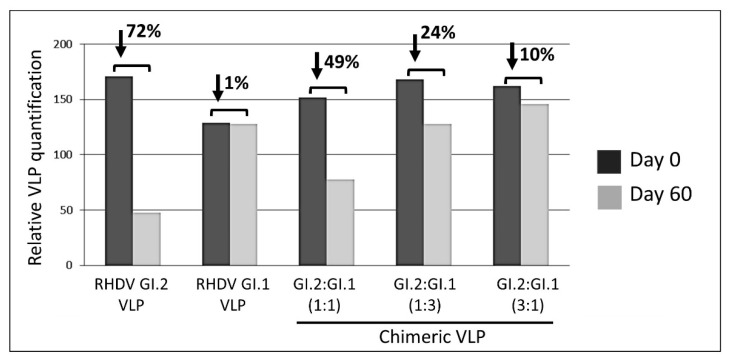
Stability after 2 months of storage of VLPs obtained with individual VP60 proteins and that obtained by co-infection with the two recombinant baculoviruses using different proportions as determined SDS-PAGE and by electron microscopy. RHDV GI.2-derived VLPs were not stable, while chimeric VLPs obtained at a RHDV GI.2/RHDV GI.1 ratio of 3:1 were similar in stability to the highly stable RHDV GI.1-derived VLPs.

To determine the consequences of the co-infection conditions on the chimeric VLP stability, we compared the VLP stability after 2 months of storage at 4 °C. For this analysis, VLPs were produced using GI.1/GI.2-derived particles at baculovirus infection ratios of 1:1, 1:3, and 3:1. In parallel, we performed the same analysis using GI.2 (N11-)- and GI.1 (AST)-derived VLPs produced independently. After this storage period, we observed a dramatic degradation (>70%) of GI.2 (N11)-derived VLPs, indicative of their low stability (Figure 4). This rapid degradation was coincident with the high percentage of T1 structures observed (Figure 1B). However, the GI.1 (AST89)-derived VLPs were highly stable, with very little degradation observed after a long period of storage. Interestingly, the different baculovirus ratios used in this experiment were crucial in VLP stability. The most stable VLPs were obtained at the GI.2 (N11)/GI.1 (AST89) ratio of 3:1, and stability decreased at a ratio of 1:3 and to a greater extent at the 1:1 ratio (Figure 4). In all cases, the stability of chimeric VLPs was higher than GI.2 (N11)-derived VLP, indicating that GI.1 (AST89) VP60 stabilises the self-assembled structure.

### 3.3. Chimeric VLPs Protect Rabbits against a Lethal Challenge with RHDV GI.1 and RHDV GI.2 Viruses

To analyse the protection conferred by the chimeric VLPs against the two predominant RHDV serotypes, we used a mixture of GI.2 (N11-) and GI.1 (AST89)-derived VLPs at a single previously determined protective dose and two doses of chimeric VLPs to immunise rabbits. As expected, the mixture of 5 µg of both VLPs induced protection against a lethal dose of RHDV GI.2 virus, showing no interference of the GI.1 (AST89)-derived VLP in this protection. Two doses of chimeric VLPs of 20 and 40 µg were also used to determine the protection ratios against RHDV GI.2, and only 40 µg was used to protect against an RHDV GI.1 virus. A high protection rate of 80% against RHDV GI.2 was achieved with the 20 µg dose of chimeric VLP, and a rate of 100% was achieved with a dose of 40 µg against both lethal viruses (Table 2), demonstrating that chimeric VLPs can be used as an efficacious vaccine covering all predominant circulating RHDV viruses. Virus genome detection was only positive in the rabbit that succumbed to the infection challenge (3.06 × 10^5^ viral RNA copies per microliter—one rabbit in group B), indicating a solid protection conferred by the chimeric VLPs. The negative control groups showed 80% mortality after infection with RHDV GI.1 or RHDV GI.2 viruses and all animals were positive for virus genome detection. Viral RNA detection ranged between 6.03 × 10^5^ and 5.81 × 10^6^ copies per microliter for animals in group A.

**Figure 5:**
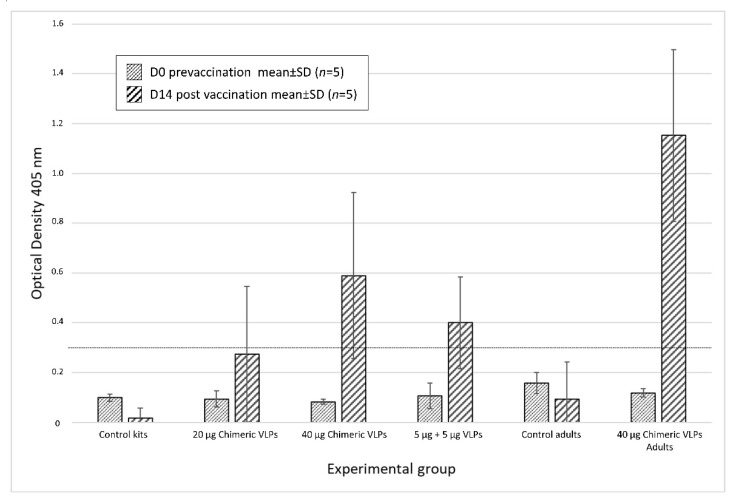
Detection of anti-RHDV antibodies in rabbit sera pre- (D0) and post vaccination (D 14) by ELISA. The graph shows optical density (450 nm) of sera versus mean ± SD (*n* = 5) from the four groups of rabbits challenged with RHDV GI.2 (kits of 30 d of age) and two groups (adults 60 d) challenged with GI.1. Sera were analysed using the commercial ELISA a cut-off value of 0.3 is indicated with the dotted line.

Antibodies generated against RHDV were analysed using a commercial ELISA from sera extracted from all rabbits on day 0 (pre-vaccination) and from surviving animals on day 14 post vaccination (7 days post challenge). As expected, all sera were negative for anti-RHDV antibodies on day 0. Seven days post challenge, the majority of surviving animals elicited an immune response as indicated by rising Ab levels (Figure 5). Overall, 40% of kits receiving the 20 µg dose of chimeric VLPs were positive and this rose to 60% for kits that received the 40 µg dose. Although not reaching the cut-off point as defined by this ELISA, the remaining animals in this group showed increased levels of Abs when compared to controls. In total, 80% of rabbits that received a mixture of non-chimeric VLPs were positive for anti-RHDV antibodies 14 days post vaccination. The highest levels of anti-RHDV antibodies were detected in adult rabbits vaccinated with 40 µg of chimeric VLPs, with 100% of this group demonstrating seroconversion and that all were protected against challenge.

## 4. Discussion

Development of efficacious recombinant subunit vaccines is essential, especially for causal agents for which a cell culture capable of supporting its propagation has not been identified. This is the case for RHDV, which is the causative agent of one of the most economically important diseases in rabbits worldwide. Commercially available vaccines are still traditional inactivated viruses collected from organs of artificially infected rabbits, which is unacceptable from both the safety and ethical points of view. The production of a new-generation vaccine against RHDV needs to meet two main requirements: first, cost-efficiency in production to make it commercially viable, and second, the vaccine has to induce a broad protection against RHDV GI.1 and RHDV GI.2 viruses as cross-protection is only partial at best [39,40].

Virus-like particles have received considerable attention due to their potential application in human and veterinary vaccines against infectious diseases. VLPs are highly immunogenic and are extremely safe. In the case of RHDV, the capsid protein forms characteristic VLPs when expressed in different systems as for the other members of the *Caliciviridae*. The productivity of this protein is generally good in most studied systems, but the development of a subunit vaccine based on the VLP that this protein forms is commercially difficult for the production and regulatory-associated costs, considering the limited number of vaccine doses applied in the domestic rabbit population worldwide. In addition, any vaccine against this virus has to be dual because of the potential of two circulating virus serotypes today, RHDV GI.1 and RHDV GI.2. In the present work, we attempted to produce RHDV VLPs in one of the most cost-efficient methods developed to date based on the use of *Trichoplusia ni* insect chrysalises (CrisBio technology), and also to develop a single production process (upstream and downstream) of a chimeric VLP-based vaccine containing the critical antigens from both predominant RHDVs circulating today.

RHDV has been previously described as a versatile platform for foreign B-cell epitope display, inducing protective humoral immune responses [41]. It has also been described that it is possible to incorporate antigenic determinants from multiple strains into a single genetic background, generating a chimeric norovirus VLP [42]. Norovirus VLPs have also been used to display large antigens on their surface by bioengineering norovirus capsid shell (S) and protruding (P) domains [43]. However, as far as we know, this is the first description in a virus of this type of a combination of two different capsid proteins in the same VLP. The chimeric nature of the VLPs produced in this system following baculovirus co-infections was confirmed using a serotype-specific MAb capture and detection in ELISA and dot blot assays. The proportion of serotype-specific VP60 incorporation could be manipulated using a varying MOI during production, adding a novel aspect to this procedure.

In the absence of a widely available tissue culture system that can sustain replication of human noroviruses, VLPs have been used as a surrogate to study the capsid’s structural features and as immunogens to elicit protective humoral responses [44]. The most advanced norovirus vaccine candidate is a bivalent formulation comprising a mixture of GI.1 and GII.4 VLPs, administered intramuscularly [45]. However, its efficacy and durability have been limited. It has been shown that norovirus GI.1 VLPs are unstable and contain a substantial fraction of dissociated VLP components. Broadly reactive, non-neutralising antibodies are isolated from vaccinated donors bound to the dissociated components, but not to the intact VLPs. We have also observed that RHDVGI.2 suffers a similar dissociation characteristic that may compromise the efficacy of future VLP-based vaccines against this virus subtype. On the other hand, for norovirus VLPs, it was possible to stabilise the VLP by engineering of interprotomer disulphide bonds within the shell domain [46]; we observed that through co-expressing the VP60 proteins from RHDV GI.1 and RHDV GI.2, we were able to obtain the same VLP stabilisation effect.

RHDV GI.1 VLPs have been previously produced in *Trichoplusia ni* larvae [12] and pupae [35]. Recently, a bivalent vaccine against RHDV GI.1 and RHDV GI.2 received the EMA positive opinion and will be commercialised in the following months by the Italian company Fatro. This vaccine combines VLPs derived from the two virus subtypes and will be produced by CrisBio technology. This technology platform makes possible the production of subunit vaccines processed similarly as in cultured insect cells but in a much more cost-efficient manner. CrisBio is extremely efficient in producing different kind of proteins including those that self-assemble to form VLPs [35]. The production in insects, particularly insect pupae, allows for the substitution of steel tanks where the cells are cultivated using natural bioreactors, namely the insect chrysalises, containing millions of cells in perfect physiological conditions ready to be programmed for production by baculovirus vectors. *Trichoplusia ni* Lepidoptera is highly susceptible to AcMNPV-derived vectors, and therefore, any common baculovirus can be used for industrial or experimental production. This technology is also linearly scalable, facilitating their implementation for large quantities of vaccine doses in a record time.

In the present work, we also used the TopBac expression cassette to generate the baculovirus vectors [36,37]. The resulting baculoviruses increase productivities up to four times, also considerably reducing the production costs.

## 5. Conclusions

In the present work, we demonstrated the feasibility of producing a vaccine against RHDV that incorporates two different capsid proteins derived from two virus subtypes and confers broad protection in immunised rabbits. The chimeric RHDV VLPs were produced in a single upstream and downstream production process, significatively reducing the complexity of a binary vaccine. In addition, the combination of TopBac baculovirus vector and CrisBio technology allows for a cost-efficient production required for a vaccine directed to domestic rabbits to make it commercially viable.

## Figures and Tables

**Table 1:** Experimental design.

Group	Age	Vaccination	Challenge
A	30 d	PBS	RHDVGI.2
B	30 d	20 µg Chimeric VLPs	RHDVGI.2
C	30 d	40 µg Chimeric VLPs	RHDVGI.2
D	30 d	5 µg RHDVGI.2 VLPs + 5 µg RHDVGI.1 VLPs	RHDVGI.2
E	60 d	PBS	RHDVGI.1
F	60 d	40 µg Chimeric VLPs	RHDVGI.1

Abbreviations: PBS Phosphate buffered saline; RHDV Rabbit haemorrhagic disease virus; VLP virus-like particle.

**Table 2:** Rabbit protection results obtained with different vaccine formulations after challenge of vaccinated rabbits with the two RHDV serotypes.

Group	Vaccination	Virus Challenge	Mortality	Virus Genome Detection
A	PBS	RHDVGI.2	4/5	5/5
B	20 µg chimeric VLP	RHDVGI.2	1/5	1/5
C	40 µg chimeric VLP	RHDVGI.2	0/5	0/5
D	5 µg RHDVGI.1 VLP +5 µg RHDVGI.2 VLP	RHDVGI.2	0/5	0/5
E	PBS	RHDVGI.1	4/5	nd
F	40 µg chimeric VLP	RHDVGI.1	0/5	nd

Abbreviations: PBS Phosphate buffered saline; RHDV Rabbit haemorrhagic disease virus; VLP virus-like particle.

## Supplementary Materials

The following are available online at https://www.mdpi.com/article/10.3390/vaccines9091005/s1, Figure S1: Full uncropped blot of SDS-PAGE gels resolving the extracts from infected pupae with individual baculovirus and stained by Coomassie blue, Figure S2: Full uncropped dotblot obtained in the different assays.

## References

[1] Cooke B.D. (2002). Rabbit haemorrhagic disease: Field epidemiology and the management of wild rabbit populations. OIE Rev. Sci. Tech..

[2] Moss S.R., Turner S.L., Trout R.C., White P.J., Hudson P., Desai A., Armesto M., Forrester N.L., Gould E.A. (2002). Molecular epidemiology of Rabbit haemorrhagic disease virus. J. Gen. Virol..

[3] Ohlinger V.F., Haas B., Meyers G., Weiland F., Thiel H.J. (1990). Identification and characterization of the virus causing rabbit hemorrhagic disease. J. Virol..

[4] Cheng Y., Chen Z., Li C., Meng C., Wu R., Liu G. (2013). Protective immune responses in rabbits induced by a suicidal DNA vaccine of the VP60 gene of rabbit hemorrhagic disease virus. Antivir. Res..

[5] Parra F., Prieto M. (1990). Purification and characterization of a calicivirus as the causative agent of a lethal hemorrhagic disease in rabbits. J. Virol..

[6] Meyers G., Wirblich C., Thiel H. (1991). Rabbit hemorrhagic disease virus-molecular cloning and nucleotide sequencing of a calicivirus genome. Virology.

[7] Meyers G., Wirblich C., Thiel H.J., Thumfart J.O. (2000). Rabbit hemorrhagic disease virus: Genome organization and polyprotein processing of a calicivirus studied after transient expression of cDNA constructs. Virology.

[8] Boga J., Marín M., Casais R., Prieto M., Parra F. (1992). In vitro translation of a subgenomic mRNA from purified virions of the Spanish field isolate AST/89 of Rabbit Hemorrhagic Disease Virus (RHDV). Virus Res..

[9] Parra F., Boga J.A., Marin M., Casais R. (1993). The amino terminal sequence of VP60 from rabbit hemorrhagic disease virus supports its putative subgenomic origin. Virus Res..

[10] Prasad B.V.V., Matson D.O., Smith A.W. (1994). Three-dimensional Structure of Calicivirus. J. Mol. Biol..

[11] Villares J.A. (1991). Viral haemorrhagic disease of rabbits: Vaccination and immune response. Rev. Sei. Tech. Off. Int. Epiz..

[12] Pérez-Filgueira D., Resino-Talavan P., Cubillos-Zapata C., Angulo I., Barderas M., Barcena J., Escribano J. (2007). Development of a low-cost, insect larvae-derived recombinant subunit vaccine against RHDV. Virology.

[13] Kushnir N., Streatfield S.J., Yusibov V. (2012). Virus-like particles as a highly efficient vaccine platform: Diversity of targets and production systems and advances in clinical development. Vaccine.

[14] Grgacic E.V.L., Anderson D.A. (2006). Virus-like particles: Passport to immune recognition. Methods.

[15] Deml L., Speth C., Dierich M.P., Wolf H., Wagner R. (2005). Recombinant HIV-1 Pr55gag virus-like particles: Potent stimulators of innate and acquired immune responses. Mol. Immunol..

[16] Chackerian B. (2007). Virus-like particles: Flexible platforms for vaccine development. Expert Rev. Vaccines.

[17] Kerr P.J., Kitchen A., Holmes E.C. (2009). Origin and Phylodynamics of Rabbit Hemorrhagic Disease Virus. J. Virol..

[18] McIntosh M.T., Behan S.C., Mohamed F.M., Lu Z., Moran E.K., Burrage T.G., Neilan J.G., Ward G.B., Botti G., Capucci L. (2007). A pandemic strain of calicivirus threatens rabbit industries in the Americas. Virol. J..

[19] Le Pendu J., Abrantes J., Bertagnoli S., Guitton J.-S., Le Gall-Reculé G., Lopes A., Marchandeau S., Alda F., Almeida T., Célio A.P. (2017). Proposal for a unified classification system and nomenclature of lagoviruses. J. Gen. Virol..

[20] Dalton K.P., Nicieza I., Abrantes J., Esteves P., Parra F. (2014). Spread of new variant RHDV in domestic rabbits on the Iberian Peninsula. Vet. Microbiol..

[21] Dalton K.P., Nicieza I., Balseiro A., Muguerza M.A., Rosell J.M., Casais R., Álvarez L., Parra F. (2012). Variant rabbit hemorrhagic disease virus in young rabbits, Spain. Emerg. Infect. Dis..

[22] Le Gall-Reculé G., Zwingelstein F., Boucher S., Le Normand B., Plassiart G., Portejoie Y., Decors A., Bertagnoli S., Guérin J.-L., Marchandeau S. (2011). Detection of a new variant of rabbit haemorrhagic disease virus in France. Vet. Rec..

[23] Capucci L., Cavadini P., Schiavitto M., Lombardi G., Lavazza A. (2017). Increased pathogenicity in rabbit haemorrhagic disease virus type 2 (RHDV2). Vet. Rec..

[24] Boga J.A., Casais R., Marin M.S., Martin-Alonso J.M., Cármenes R.S., Prieto M., Parra F. (1994). Molecular cloning, sequencing and expression in Escherichia coli of the capsid protein gene from rabbit haemorrhagic disease virus (Spanish isolate AST/89). J. Gen. Virol..

[25] Boga J.A., Alonso J.M.M., Casais R., Parra F. (1997). A single dose immunization with rabbit haemorrhagic disease virus major capsid protein produced in Saccharomyces cerevisiae induces protection. J. Gen. Virol..

[26] Castañón S., Marín M.S., Martín-Alonso J.M., Boga J.A., Casais R., Humara J.M., Ordás R.J., Parra F. (1999). Immunization with Potato Plants Expressing VP60 Protein Protects against Rabbit Hemorrhagic Disease Virus. J. Virol..

[27] Fernández-Fernández M.R., Mouriño M., Rivera J., Rodríguez F., Plana-Durán J., García J.A. (2001). Protection of Rabbits against Rabbit Hemorrhagic Disease Virus by Immunization with the VP60 Protein Expressed in Plants with a Potyvirus-Based Vector. Virology.

[28] Bertagnoli S., Gelfi J., Le Gall G., Boilletot E., Vautherot J.F., Rasschaert D., Laurent S., Petit F., Boucraut-Baralon C., Milon A. (1996). Protection against myxomatosis and rabbit viral hemorrhagic disease with recombinant myxoma viruses expressing rabbit hemorrhagic disease virus capsid protein. J. Virol..

[29] Bertagnoli S., Gelfi J., Petit F., Vautherot J., Rasschaert D., Laurent S., Le Gall G., Boilletot E., Chantal J., Boucraut-Baralon C. (1996). Protection of rabbits against rabbit viral haemorrhagic disease with a vaccinia-RHDV recombinant virus. Vaccine.

[30] Fischer L. (1997). A recombinant canarypox virus protects rabbits against a lethal rabbit hemorrhagic disease virus (RHDV) challenge. Vaccine.

[31] Laurent S., Vautherot J., Madelaine O., le Gall G., Rasschaertl D. (1994). Recombinant Rabbit Hemorrhagic Disease Virus Capsid Protein Expressed in Baculovirus Self-Assembles into Viruslike Particles and Induces Protection. J. Virol..

[32] Marín M.S., Alonso J.M., García L.I.P.O., Boga J.A., Argüello-Villares J., Casais R., Venugopal K., Jiang W., Gould E.A., Parra F. (1995). Immunogenic properties of rabbit haemorrhagic disease virus structural protein VP60 expressed by a recombinant baculovirus: An efficient vaccine. Virus Res..

[33] Nagesha H.S., Wang L.F., Hyatt A.D., Morrissy C.J., Lenghaus C., Westbury H.A. (1995). Self-assembly, antigenicity, and immunogenicity of the rabbit haemorrhagic disease virus (Czechoslovakian strain V-351) capsid protein expressed in baculovirus. Arch. Virol..

[34] Sibilia M., Boniotti M.B., Angoscini P., Capucci L., Rossi C. (1995). Two Independent Pathways of Expression Lead to Self-Assembly of the Rabbit Hemorrhagic Disease Virus Capsid Protein. J. Virol..

[35] Escribano J.M., Cid M., Reytor E., Alvarado C., Nuñez M.C., Martínez-Pulgarín S., Dalton R.M. (2020). Chrysalises as natural production units for recombinant subunit vaccines. J. Biotechnol..

[36] López-Vidal J., Gómez-Sebastián S., Barcena J., Nuñez M.D.C., Martínez-Alonso D., Dudognon B., Guijarro E., Escribano J.M. (2015). Improved production efficiency of virus-like particles by the baculovirus expression vector system. PLoS ONE.

[37] Gomez-Sebastian S., López-Vidal J., Escribano J.M. (2014). Significant productivity improvement of the baculovirus expression vector system by engineering a novel expression cassette. PLoS ONE.

[38] Dalton K.P., Arnal J.L., Benito A.A., Chacón G., Alonso J.M.M., Parra F. (2018). Conventional and real time RT-PCR assays for the detection and differentiation of variant rabbit hemorrhagic disease virus (RHDVb) and its recombinants. J. Virol. Methods.

[39] Calvete C., Mendoza M., Alcaraz A., Sarto M.P., Jiménez-de-Bagüéss M.P., Calvo A.J., Monroy F., Calvo J.H. (2018). Rabbit haemorrhagic disease: Cross-protection and comparative pathogenicity of GI.2/RHDV2/b and GI.1b/RHDV lagoviruses in a challenge trial. Vet. Microbiol..

[40] Mahar J.E., Hall R.N., Peacock D., Kovaliski J., Piper M., Mourant R., Huang N., Campbell S., Gu X., Read A. (2018). Rabbit Hemorrhagic Disease Virus 2 (RHDV2; GI.2) Is Replacing Endemic Strains of RHDV in the Australian Landscape within 18 Months of Its Arrival. J. Virol..

[41] Moreno N., Mena I., Angulo I., Gómez Y., Crisci E., Montoya M., Caston J., Blanco E., Bárcena J. (2016). Rabbit hemorrhagic disease virus capsid, a versatile platform for foreign B-cell epitope display inducing protective humoral immune responses. Sci. Rep..

[42] Debbink K., Lindesmith L.C., Donaldson E.F., Swanstrom J., Baric R.S. (2014). Chimeric GII.4 Norovirus Virus-Like-Particle-Based Vaccines Induce Broadly Blocking Immune Responses. J. Virol..

[43] Tzeyung A.S., Shadab, Bhattamisra S.K., Madheswaran T., Alhakamy N.A., Aldawsari H.M., Radhakrishnan A.K. (2019). Fabrication, Optimization, and Evaluation of Rotigotine-Loaded Chitosan Nanoparticles for Nose-To-Brain Delivery. Pharmaceutics.

[44] Cortes-Penfield N.W., Ramani S., Estes M.K., Atmar R.L. (2017). Prospects and Challenges in the Development of a Norovirus Vaccine. Clin. Ther..

[45] Treanor J.J., Atmar R.L., Frey S.E., Gormley R., Chen W.H., Ferreira J., Goodwin R., Borkowski A., Clemens R., Mendelman P.M. (2014). A Novel Intramuscular Bivalent Norovirus Virus-Like Particle Vaccine Candidate-Reactogenicity, Safety, and Immunogenicity in a Phase 1 Trial in Healthy Adults. J. Infect. Dis..

[46] Verardi R., Lindesmith L.C., Tsybovsky Y., Gorman J., Chuang G.-Y., Edwards C.E., Brewer-Jensen P.D., Mallory M.L., Ou L., Schön A. (2020). Disulfide stabilization of human norovirus GI.1 virus-like particles focuses immune response toward blockade epitopes. NPJ Vaccines.

