# Supplementary material for: Chimeric VLPs Bearing VP60 from Two Serotypes of Rabbit Haemorrhagic Disease Virus Are Protective against Both Viruses
Source: Vaccines (Basel). 2021 Sep 9;9(9):1005. doi: 10.3390/vaccines9091005 (PMC8472679; doi:10.3390/vaccines9091005)
Supplement: Supplementary file 1 [file vaccines-09-01005-s001.zip › vaccines-1348291-supplementary.pdf]

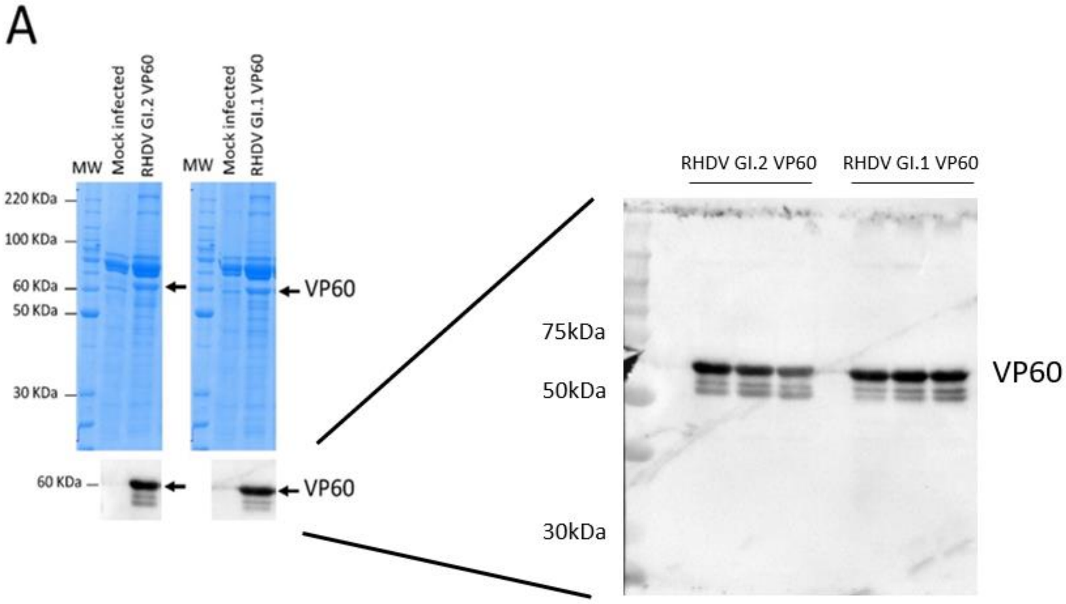

Figure S1. Full uncropped blot of SDS-PAGE gels resolving the extracts from infected pupae with individual baculovirus and stained by Coomassie blue.

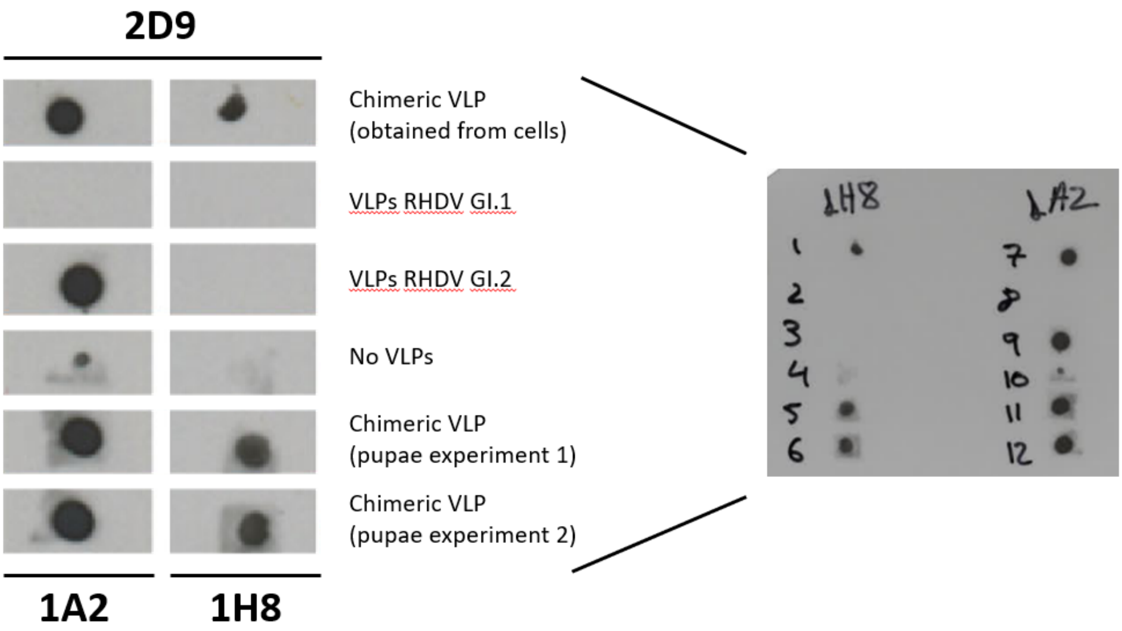

Figure S2. Full uncropped dotblot obtained in the different assays.
